# Searching for new molecular markers for cells obtained from abdominal aortic aneurysm

**DOI:** 10.1007/s13353-021-00641-4

**Published:** 2021-06-02

**Authors:** Marta Lesiak, Aleksandra Augusciak-Duma, Karolina L. Stepien, Agnieszka Fus-Kujawa, Malwina Botor, Aleksander L. Sieron

**Affiliations:** Department of Molecular Biology, Faculty of Medical Sciences in Katowice, Medical University of Silesia, Medyków 18 Street, Bldg. C-1, 40-752 Katowice, Poland

**Keywords:** Abdominal aortic aneurysm (AAA), Gene expression, Layers of AAA, Cytometry useful markers

## Abstract

The aim of the study was to investigate specific potential markers for cells obtained from three layers of human AAA divided into three segments along the AAA based on morphological differences. The isolated cells were compared to control commercial cell types from healthy human abdominal aortas. For each type of aortic layer, three specimens from 6 patients were compared. Total RNA was isolated from 36 cell cultures for gene expression profiling and potential new cytometry markers were typed. Isolated cells were analyzed by flow cytometry by using fluorochrome-conjugated antibodies to markers: CNN1, MYH10, ENG, ICAM2, and TEK. The relative expression of 45 genes in primary cell cultures and control lines was analyzed. Statistically significant differences were found in the expression of most of the analyzed genes between individual layers and control lines. Based on relative expression, antibodies were selected for flow cytometry. Gene expression profiles allowed to select new potential cytometry markers: CNN1, MYH10, MYOCD, ENG, ICAM2, TEK. However, none of the tested markers seems to be optimal and characteristic for a specific layer of AAA.

**Supplementary Information:**

The online version contains supplementary material available at 10.1007/s13353-021-00641-4.

## Introduction

An abdominal aortic aneurysm (AAA) is a pathological dilatation of the infrarenal aorta. It develops as a result of abdominal aortic wall dilation, which might lead to blood vessel rupture very often causing the patient’s death (Davis et al. 2015). AAA is a multifactorial disease and many risk factors which can lead to AAA development have been identified. The most important of them are smoking, older age, male gender (Kuivaniemi et al. 2015; Khan et al. 2015; Golledge et al. 2006), oxidative stress, and inflammation of the aortic wall (Davis et al. 2015; McCormick et al.^2007^), as well as hypertension and dyslipidemia (Golledge et al. 2006; Sakalihasan et al. 2018). An abdominal aortic aneurysm is the cause of death in adults as a result of aortic rupture and the only treatment for AAA is open or endovascular surgical repair (Golledge 2019). Because no drug therapy is available for AAA, it is essential to understand its pathogenesis and know the specific markers involved in the development of AAA.

Anatomically normal abdominal aorta consists of three wall layers: external, middle, and internal. In the external layer, which is called tunica adventitia, fibroblasts are the main cell type (Komutrattananont et al. 2019). Fibroblasts in there produce collagen types I and III, the major components of ECM (Myllyharju and Kivirikko 2001).

Middle layer (*tunica media*) is mainly formed by smooth muscle cells (SMC), which maintain the vessel’s structure (Komutrattananont et al. 2019; Riches et al. 2013), and regulate vascular tone and diameter through contraction by controlling blood pressure and blood flow distribution (Owens et al. 2004). In the internal layer (tunica intima), endothelial cells (EC) are the main cell type (Komutrattananont et al. 2019; Mai et al. 2013). These cells regulate tissue-fluid homeostasis, transport of nutrients, and migration of blood cells across the barrier (Komarova et al. 2017).

The process of aneurysm formation leads to apoptosis and a change in the phenotype of the cells that make up the individual layers of the aorta (Lesiak et al. unpublished work).

Determining the phenotype of cell forming layers of the aorta and learning specific markers would allow a better understanding of the process of AAA formation. Lindquist Liljeqvist showed that in the tunica media is upregulation of gene sets related to extracellular matrix (ECM) disassembly, angiogenesis, apoptosis, complement, coagulation, and phagocytosis, as well as downregulation of those related to differentiation, development, and contraction of muscle cell, cell adhesion, and assembly of ECM and elastic fibers. The adventitia showed downregulation of gene sets associated with metal ion response, adipogenesis, and cholesterol homeostasis (Lindquist Liljeqvist et al. 2020).

Also, Ziaja D. research on the expression of cytokines in different parts of the aorta showed different intensities of the destructive process in the aortic wall in its middle part.

He has reported that the intensity of the destructive processes taking place in the AAA wall was in its central layer of the middle segment of AAA. His preliminary studies showed that the individual parts of the aneurysm differ in many chemical, biochemical, physical, and molecular parameters (Ziaja 2013).

Therefore, for the reported here project we hypothesized that the cells from each layer in the separate segments should differ in their gene expression patterns both between each other and control cells. Thus, the current study aimed on detecting novel molecular markers for cells obtained from different parts of AAA what should be useful for better understanding the molecular processes ongoing in AAA.

We investigated specific potential markers characteristic for cells from the layers of AAA. For this purpose, RNA isolated from AAA cells was subjected to gene expression analysis and potential markers: CNN1, MYH10, MYOCD, ENG, ICAM2, and TEK were typed for cytometry analysis.

## Materials and methods

### Patients characteristic

Six patients (5 men and 1 woman) with abdominal aortic aneurysm after surgical treatment were included in the study. The average age of the patients was 69.5 years (Table 1).

**Table 1:** Summary of patients’ characteristics whom were donors of samples for cell and mRNA isolation

Patient no	Sex	Age (years)
T14	M	66
T15	F	75
T20	M	58
T23	M	82
T25	M	65
T29	M	71
Mean ± S.D		69.5 ± 8.4

*M*, male; *F*, female; *S.D.*, standard deviation.

Exclusion of the patients from the study was based on the following criteria: (a) chronic obstructive pulmonary disease; (b) diabetes; (c) creatinine level > 1.0; (d) reconstruction of coronary vessels and thoracic aorta; (e) reconstruction of carotid artery; (f) with diagnosed generalized atherosclerosis; (g) with AAA’s family history or inherited cardiovascular syndromes; (h) whom were unable to sign informed consent for surgical treatment. All surgical procedures were performed in the planned mode. Briefly, the material was collected intraoperatively in General and Vascular Surgery Department in Katowice-Ochojec, Poland, and secured immediately in the operating room to prepare sterile 50 mL tubes filled with 25 mL of Dulbecco’s Modified Eagle’s Medium (Gibco, Grand Island, NY, USA) supplemented with glucose (4.5 mg/mL) (high-glucose DMEM), penicillin (10,000 U/ml), streptomycin (10 mg/ml) and amphotericin B (25 µg/ml) (PAA Laboratories, Pasching, Austria) and immediately delivered to the cell culture laboratory. Specifically, all treatments were started at 8:30 AM, the biopsies were taken after implantation, between 9:00 and 10:00 AM. The courier picked up the tube no later than at 11:00 AM. The sample was in the cell culture facility of the Department of Molecular Biology and Genetics, Medical University in Katowice, Poland, within the next 30 min. The research protocol was approved by the Bioethics Committee of the Silesian Medical University in Katowice, no. KNW/0022/KB1/55/14 issued on June 17, 2014, and extension to KNW/0022/KB1/55/1/14/17, issued on June 27, 2017 (Ziaja 2013).

### Isolation of cells from abdominal aortic aneurysms

**Fig. 1:**
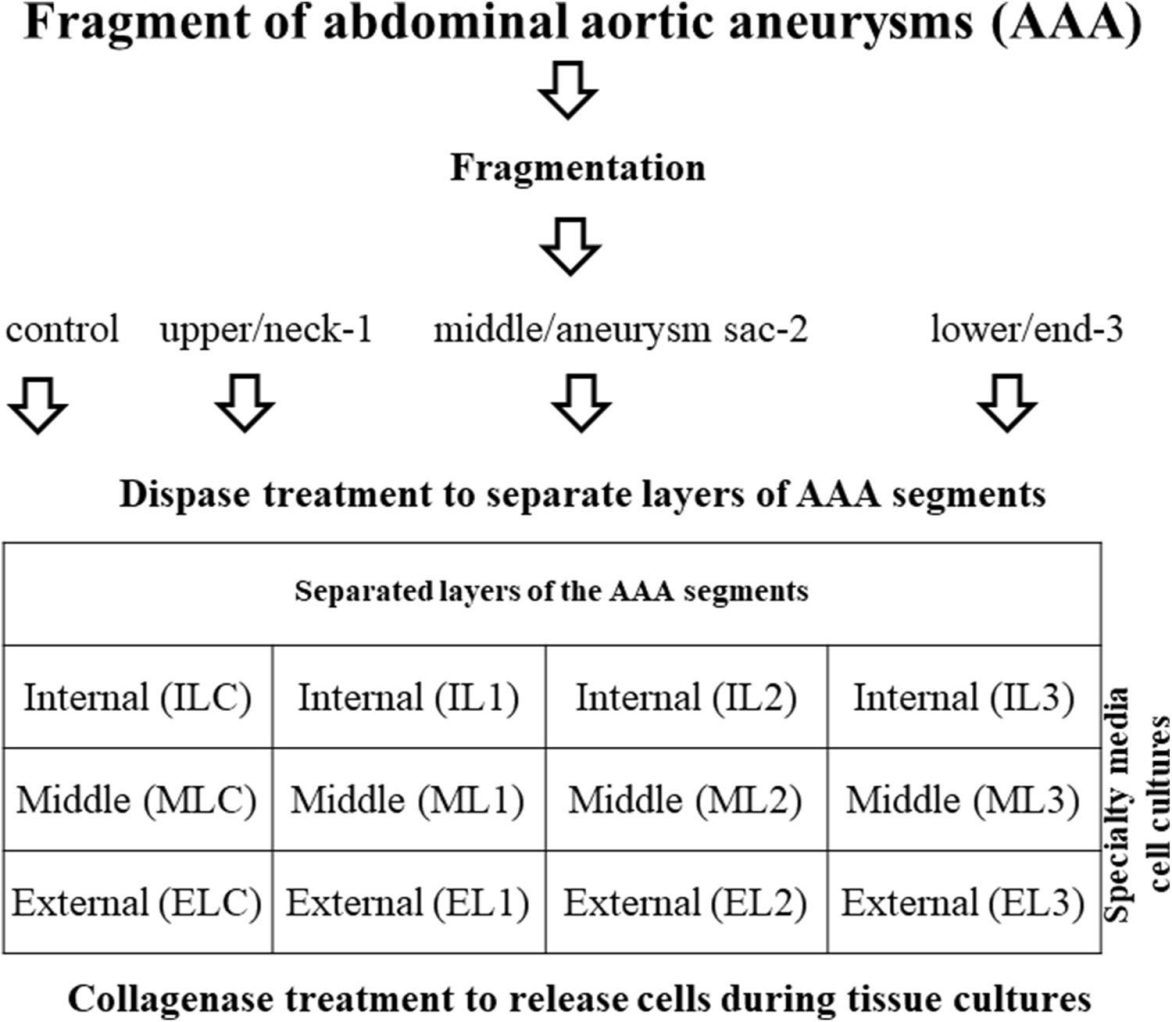
Schematic of isolation of cells specific for wall layers of AAA segments. Segments of abdominal aortic aneurysms (AAA) after fragmentation and enzyme treatment were split into 3 layers of the wall: tunica intima (internal layer—IL), tunica media (middle layer—ML) and tunica adventitia (external layer—EL)

Fragments of an abdominal aortic aneurysm (AAA) around 50 mm long and 18 mm in diameter were obtained from patients following surgery. The AAAs’ fragments were removed otherwise they would be discarded. Collected samples were processed immediately upon arrival to the cell culture facility. Under sterile conditions, AAA fragments were rinsed 3 times with phosphate-buffered saline (PBS) (PAA Laboratories, Pasching, Austria). The sample top end marked by the surgeon as a margin (~ 2 mm) was cut out and assumed to be a control. The remaining fragment was divided into 3 equal length fragments. The divisions were reflecting the morphology of the aneurysm. In the aneurysm, the neck, aneurysm sac and its end section are distinguished. The end segment reaches, and sometimes covers, the aortic bifurcation or iliac arteries. The neck segment was defined as the upper one, 1; the aneurysm bag as the middle one, 2; and the end segment as the lower one, 3 (Fig. 1). All dissected fragments were subjected to incubation in the presence of dispase II (Gibco, Grand Island, NY, USA) (2.4 U/mL) in PBS for 30 min at 37 °C. Subsequently, the longitudinal fragments were split into 3 layers of the wall: tunica intima (internal layer—IL), tunica media (middle layer—ML), and tunica adventitia (external layer—EL) (Fig. 1). Subsequently, 2/3 of each layer were minced to very fine pieces and subjected to incubation in the presence of collagenase type I (3 mg/mL) (Gibco, Grand Island, NY, USA) for 30 min at 37 °C. Collagenase type I was inactivated by the addition of high-glucose DMEM supplemented with 10% of fetal bovine serum (FBS) (Gibco, Grand Island, NY, USA), antibiotics and antimycotic as before. A total of 3 tissue cell cultures from each aneurysm sample were established with culture media dedicated to aortal: endothelial cells, smooth muscle cells and fibroblasts (Ziaja 2013).

A complete set of 9 samples was obtained only from patient T29 only. For the remaining patients, the samples were combined to form a total of 3 sets, respectively, for the inner, middle, and external layer of the aorta. These sets were combined and used for further analyses of gene expression. As a control, commercial cultures for each layer were used (Table 2).

**Table 2:** Summary of the layers and patients

Patient no	Internal layer (IL)			Middle layer (ML)			External layer (EL)		
	Tunica intima			Tunica media			Tunica adventitia		
	Vertical segments			Vertical segments			Vertical segments		
	IL1	IL2	IL3	ML1	ML2	ML3	EL1	EL2	EL3
T14	x	x	x	x	x	x			
T15								x	x
T20				x	x	x	x		
T23	x	x	x						
T25							x	x	x
T29	x	x	x	x	x	x	x	x	x
HAEC	x	x	x						
AoSMC				x	x	x			
AoAF							x	x	x

*x* represents the material taken from a particular layer of the patient for analysis of gene expression.

### Cell culture

The cells from patients’ IL of AAA fragments and commercial control Human Aortic Endothelial Cells (HAEC) (Lonza, Basel, Switzerland) were cultured in EGM-2 Endothelial Medium BulletKit (Lonza, Basel, Switzerland). The cells from patients’ ML of AAA fragments and commercial control human Aortic Smooth Muscle Cells (AoSMC) (Lonza, Basel, Switzerland) were cultured in SmBM-2 Smooth Muscle BulletKit (Lonza, Basel, Switzerland). The patients’ EL of AAA fragments and control cells, for them Human Aortic Adventitial Fibroblasts (AoAF) (Lonza, Basel, Switzerland) were cultured in SCGM Stromal Cell BulletKit (Lonza, Basel, Switzerland). Morphology of the cells outgrown from the cultured fragments was inspected daily using an inverted contrast-phase light microscope (Olympus, T5 SN, Japan). At the beginning, the cell cultures were conducted in 6-well plates (culture surface 9.6 cm^2^/well) at 37 °C, 95% air, and 5% CO_2_. During the first 3 days of the culture, the medium was replaced with fresh every day. Upon further culture, the media were changed every other day. At 80% of confluence, the cells were transferred from each single well of 6-well plate to a 25 cm^2^ culture flask and again to a 75 cm^2^ culture flask. Sub-confluent cells were subjected to freezing in liquid nitrogen until further study (Ziaja 2013).

### RNA analysis

Total RNA was isolated from cell culture by Zymogen Quick RNA Mini Prep (Ambion, Austin, Texas, USA). Quality and quantity evaluation was performed using a NanoDrop 2000 spectrophotometer (Thermo Fisher Scientific, Waltham, Massachusetts, U.S.A.). Total RNA (1 to 2 µg) was transcribed using a cDNA Transcriptor First Strand cDNA Synthesis Kit (Roche, Penzberg, Upper Bavaria, Germany) using random hexamers. Expression analyses with Real Time Custom Panel 384–96 (config. no 100142046; Roche) and LightCycler480 Probe Master (Roche) were performed using LightCycler480 II (Roche). The genes analyzed in this report are listed in Supplement Table 1

Total RNA of quality and at the amount required for gene expression profiling was successfully isolated from 36 cell cultures. The characteristics of the patients whose samples were used for further gene expression analysis (gender, age) are summarized in Table 1.

For each layer of the aneurysm, tissue cell cultures derived from 3 patients and from standard commercial culture for each layer were analyzed by the gene expression (Table 2) and from standard commercial culture for each layer.

### Gene expression profiling

The gene expression was analyzed using GenEx ver6 software. Raw data were subjected to normalization to the sample number followed by normalization to reference genes—GAPDH, PPIA, and RPL0 (Supplement Table 1). The last preprocessing step was filling the missing data with 0, and relative quantification was performed using the comparative threshold (Ct) method (ΔΔCt), where the relative gene expression level equals to 2^-ΔΔCt^.

### Fluorescence assisted cell flow cytometry analyses

The cells from a specific aorta layer were analyzed by using Facs Aria I instrument (Becton Dickinson, Franklin Lakes, New Jersey, USA). Fluorochrome-conjugated specific antibodies directed against a particular surface or cytoplasmic antigen were used. The cells obtained of the three layers from each segment of the aneurysm and expanded in culture were recovered following incubation with Accutase Cell Detachment Solution (Becton Dickinson, Franklin Lakes, New Jersey, USA) by centrifugation at 159 × *g* for 5 min. The samples were washed twice with PBS (PAA Laboratories, Pasching, Austria), resuspended, and counted with the use of a cell counter (Bio Rad, Hercules, California, USA). After a final collection of the cells by centrifugation before they were washed in Flow Cytometry Staining Buffer (R&D Systems, Minneapolis, MN, USA) and resuspended to a final cell density of 10^6^ cells/200 µl. Fluorochrome-conjugated antibodies against surface antigens were added for detection of ENG (CD105) (Exbio, Praha, Czech Republic), ICAM2 (CD102) (Exbio, Praha, Czech Republic) and TEK (CD202b) (BioLegend, San Diego, USA). After 30 min of incubation with the appropriate antibody at room temperature, the cells were centrifuged as before. To remove the excess of antibodies following staining, 2 mL of Flow Cytometry Staining Buffer (R&D Systems, Minneapolis, MN, USA) was added. The cells were centrifuged again and resuspended to a final cell density of 10^6^ cells in 400 µl of Flow Cytometry Staining Buffer (R&D Systems, Minneapolis, MN, USA) and analyzed with the use of Facs Aria I. AoAF cells were used as a positive control cell for the cells obtained from EL. HAEC cells were used as a positive control for the cells obtained from IL. The negative control cells without treatment were used to set the scale.

Fluorochrome-conjugated antibodies against cytoplasmic antigens were added for detection of MYH10 (Santa Cruz Biotechology, Inc., Heidelberg, Germany) and CNN1 (calponin 1 antibody) (Novusbio, Minneapolis, USA). Respectively, after recovery of the cells from the culture as already described the cells were washed twice with PBS (PAA Laboratories, Pasching, Austria) and centrifuged as before. The cells were counted with the use of a cell counter. Following centrifugation, the cells were resuspended in Flow Cytometry Fixation/Permeabilization Buffer I (R&D Systems, Minneapolis, MN, USA) and incubated at 2–8 °C for 30 min. After incubation, the cells were centrifuged at 587 × *g* for 5 min and resuspended to a final density of 10^6^ cells in 200 µl of Flow Cytometry Permeabilization/Wash Buffer I (cat. no. FC005, R&D Systems, Minneapolis, MN, USA). Fluorochrome-conjugated antibodies against cytoplasmic antigens were added to the cell suspension and incubated for 40 min in a covered ice bucket. To wash off excess antibodies following staining, 2 mL of Flow Cytometry Permeabilization/Wash Buffer I (R&D Systems, Minneapolis, MN, USA) was added. The cells were centrifuged for 5 min at 587 × *g*, resuspended in 400 µl of Flow Cytometry Staining Buffer-FC001 (R&D Systems, Minneapolis, MN, USA) and analyzed using FACS Aria I instrument. AoSMC cells were used as a control to the cells obtained from ML. The negative control cells without treatment were used to set the scale.

### Statistical analyses

Kolmogorov–Smirnov test was employed to determine if the data from the population showed a normal distribution. For data with normal distribution, the *T*-test 1-tail was engaged in analysis of the data. For the data that were not normally distributed, a nonparametric test (Mann–Whitney 1-tailed test) was used for analysis of the data. The threshold for *p*-value was set less than 0.05.

## Results

### Relative expression in standard cell cultures

We analyzed the relative expression of marker genes in cultured cells assumed as standard cells (HAEC for endothelial cells in the internal layer—IL; AoSMC for smooth muscle cells in middle layer—ML; and AoAF for fibroblasts in the external layer EL). The most important factors for accepting the gene as a potential marker for cells were the significant differences in expression between standard cells for each layer (Supplement Tables 2 and 3). From this step, we focused on the *ALCAM*, *CD40*, *CNN1*, *KRT18*, *KRT8*, *MYH10*, *MYOCD*, *ENG*, *TEK*, *CD34*, *CD70*, *CD90*, *CDH5*, *IL1R2*, *PECAM1*, and *S100A4* (Supplement Tables 2, 3, 4, 5).

(Supplement Tables 2. *T*-test *p*-values, Supplement Table 3. Mann–Whitney *p*-values, Supplement Table 4. Fold change differences between layers of AAA, Supplement Table 5. Relative expression of analyzed genes).

### Relative expression in primary cell cultures

The second stage in determining a potential marker was to analyze whether primary cultures derived from specific AAA layers gave the higher gene expression than in standard cultures, since the expression of potential markers in the primary cultures should be lower. For the internal layer, these genes are *ACTA1*, *ANGPTL4*, *CD209*, *CD68*, *CD83*, *CD90*/*THY1*, *DDR2*, *ITGA1*, *NOS3*, *S100A4SELP*, and *SMTN*. For the middle layer, these genes are *ANGPTL4*, *CD34*, *CD83*, *ICAM2*, *S100A4*, *SELP*, and *VWF*. And for the external layer: *ANGPTL4*, *C5AR1*, *CD163*, *CD1D*, *CD40*, *CD69*, *CD90*/*THY1*, *CSF1R*, *EPCAM*, *ICAM2*, *KRT18*, *KRT8*, *MYOCD*, *PECAM1*, *S100A4*, *TNFRSF8*.

The third step was to determine whether the expression of a particular potential new marker was consistent across specific layers in the three segments of AAA. Based on the gene expression, it is not possible to distinguish different cells based on a single gene because we observed the expression of all genes in all primary cell lines. Only in standard cell culture we observed the lack of expression of nine genes. *MYOCD*, *CD86*, *CD163*, *IL1R2*, and *IL2RA* were not expressed in HAEC. There was no *SMTN*, *NOS3*, *CD209*, *IL2RA* expression in the AoAF culture. And in the AoSMC culture, we observed the lack of *NOS3*, *CD1A*, *CD86,* and *IL2RA*.

**Fig. 2:**
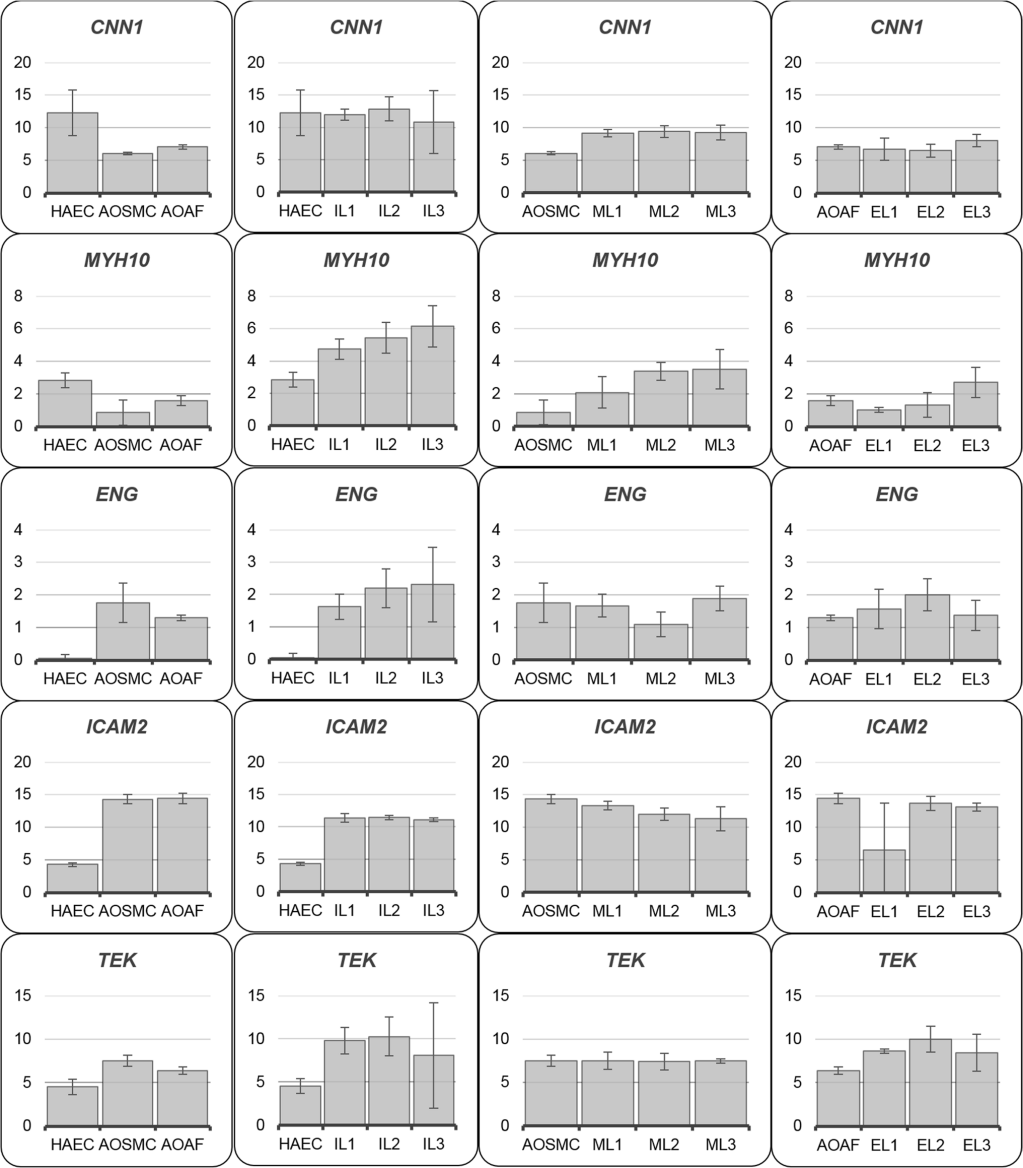
Relative expression of potential new markers. *Y* axis represents relative expression Nrel = 2^(Δ*C* normalized target − Δ*C* control), axis *X* represents expression of genes in particular cell lines

From these analyses, we suggested six potential new cytometry marker genes encoding: CNN1, MYH10, MYOCD, ENG, ICAM2 and TEK (Fig. 2). Since it was not possible to obtain antibodies for MYOCD cytometry measurements, only five proteins were analyzed.

### Phenotypic analysis of cells isolated from the layers of AAA longitudinal fragments using flow cytometry

**Fig. 3:**
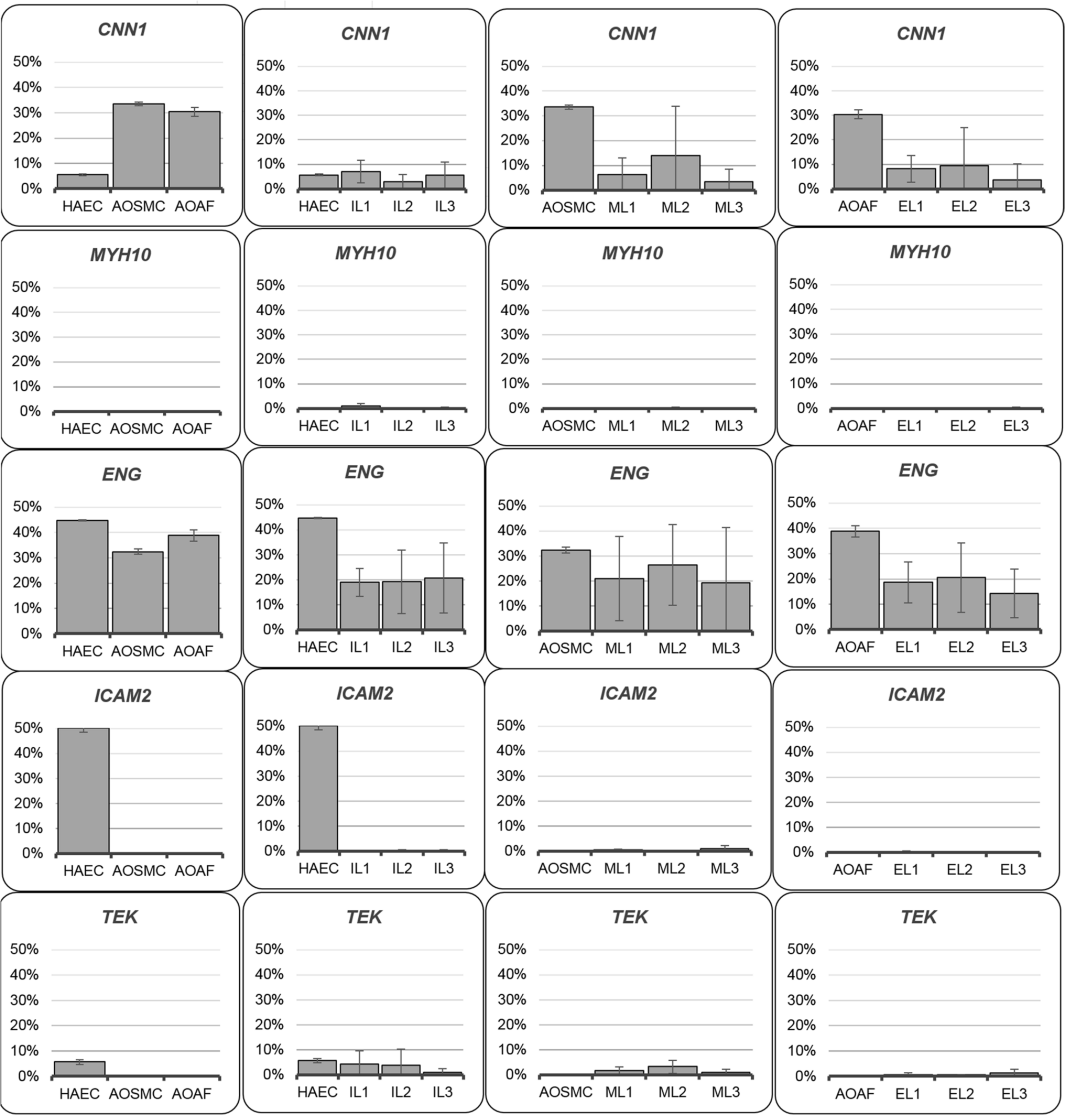
Phenotypic analysis of cells by flow cytometry. Number of positive cells for the tested marker expressed in %

Analysis of the flow cytometry data of cells obtained from AAA ILs revealed that these cells were positive for ENG, TEK, and CNN1 in comparison to control endothelial cells from the aortic wall which were positive for ENG, ICAM2, TEK and CNN1. In the control, cells with IL of the aortic wall of the AAA were 44.7% of cells with ENG, 50.2% cells with ICAM2, 5.7% of cells with TEK and 5.6% cells with CNN1. In cells isolated from ILs, 18.97%, 19.23%, and 20.8% of fragments 1, 2, and 3 were positive for ENG. Moreover, a small number of cells were positive for TEK and 4.33%, 3.87%, and 1.13% did have that marker in fragments 1, 2, and 3. Analyses obtained also that cells isolated from ILs were positive for CNN1 and 7.07%, 2.87%, and 5.53% of fragments 1, 2, and 3 were positive for CNN1 (Fig. 3).

The cells obtained from AAA MLs were positive as expected for CNN1, but also for ENG and in small numbers for TEK. In these cells, 6.37%, 13.93%, and 3.5% were positive for CNN1 in the fragments 1, 2, and 3. Many more cells were positive for ENG and it was 20.97%, 26.53%, and 19.37% in fragments 1, 2, and 3.

Only a small number of cells obtained from ML of the aortic wall did have the marker TEK and it was 1.73%, 3.3%, and 1.03% in fragments 1, 2, and 3.

In the control, cells in ML of the aortic wall of the AAA were 33.5% of cells with CNN1 and 32.4% cells with ENG (Fig. 3).

Cells isolated from EL of the aortic wall of the AAA, as expected, did not have detectable markers ICAM2 and TEK that are characteristic for cells in the IL of the aortic wall. Moreover, they were negative for MYH10. Instead, some 8.17%, 9.53%, and 3.7% of these cells were positive for CNN1 in fragments 1, 2, and 3. Many more cells were positive for ENG and it was 18.67%, 20.57%, and 14.3% in fragments 1, 2, and 3. In the control, cells with EL of the aortic wall of the AAA were 30.4% of cells with CNN1 and 38.8% cells with ENG (Fig. 3).

## Discussion

In our research, we tried to select a specific marker for cells isolated from AAA layers. As of today, there is no specific biomarker for AAA development, and molecular analysis of the affected tissue often yields conflicting results. The complex mechanisms involved in the development of aneurysms need to be investigated, but tools are lacking, or rather there are a large number of proteins that are being analyzed in experiments in the scientific and medical world. For this reason, it does not allow the results to be easily compared with the literature. Any attempt to slow the development of AAA or even heal the affected tissue must be tested not only in animal models but also in cultured cells. Obtaining primary cultures from AAA tissue is a complex and difficult task, and as a result, scientists are provided with a mixture of cells that must be identified and maintained in cell culture over a period of time (Lesiak et al. unpublished work; Ziaja 2013). The analysis of these cells without proper identification is futile. Based on the analysis of gene expression, we selected potential new cytometric markers: CNN1, MYH10, ENG, ICAM2 and TEK. We wanted to investigate one of them, which could be a specific marker for one of the AAA layers and could be useful for distinguishing cells from it.

The process of AAA formation is characterized by a change in the phenotype of cells building individual layers of the aortic wall (Lesiak et al. unpublished work). In the process of AAA formation, a change in cell phenotype (Ailawadi et al. 2009; Yuan and Wu 2018), cell apoptosis (Golledge 2019), as well as different expression of genes associated with ECM degradation have been noticed (Farrell et al. 2019; Kobayashi et al. 2002; Armstrong et al. 2002; Quintana and Taylor 2019).

Ailawadi examined the expression of smooth muscle cell marker genes, including SM22A, α-SMA, MMP-2, and MMP-9 in abdominal aneurysm tissue in comparison to control tissue (Ailawadi et al. 2009). He demonstrated a decrease in SM marker genes, which was explained by SMC apoptosis and by changes in SMC phenotype in response to changing of surrounding environment (Ailawadi et al. 2009). Lack of α-SMA positive cells in the middle layer of the aortic wall in patients with AAA was observed in comparison to control cells of aorta (Lesiak et al. unpublished work). α-SMA expressions in aortic smooth muscle cells of patients with AAA were significantly reduced (Yuan and Wu 2018).

CNN1 is a regulator of smooth muscle contractility and responsiveness to contractile activation (Liu and Jin 2016). It is an actin filament associated regulatory protein, expressed both in smooth muscle and in many nonmuscle cells (Liu and Jin 2016). In our study, the number of CNN1 positive cells was also reduced in all layers of the aorta compared to control cells. In contrast, CNN1 gene expression was surprisingly highest in HAEC control cells (difference in fold change: 74 in AoSMC and 36 in AoAF) and in inner layer cells from AAA patients. We expected it to be the highest in the middle layer (Hadi et al. 2018). The discrepancy may be due to 2D culture as this protein is involved in the regulation of the microenvironment and interaction within the ECM (Birgersdotter et al. 2005; Dozio et al. 2019).

MYH10 is one of the isoforms of nonmuscle myosin II B. It is involved in cell shape determination, cytokinesis, remodeling of the ECM, and formation of cell–cell adhesion (Heissler and Manstein 2013). MYH10 is commonly referred to as a marker of aortic smooth muscle cells and tunica intima (Wang et al. 2018). Analysis of mRNA expression in our study did not confirm this. In our study, MYH10 gene expression was the highest in HAEC compared to other control cells. MYH10 gene expression was also highest in cells from internal AAA layers. Nonmuscle myosin IIB has a significant effect on the maintenance of the cell–cell junction and is also a negative regulator of self-renewal of pluripotent stem cells (Walker et al. 2010; Newell-Litwa et al. 2015). It is therefore not surprising that the inner layer has a large amount of mRNA for this protein. It was unexpected that flow cytometric analyses did not show positive results for MYH10 cells in all layers.

Endoglin is a 180 kDa type I transmembrane glycoprotein and functions as a receptor for the ligand of the TGF-β superfamily (Schoonderwoerd et al. 2010). Endoglins regulate proliferation (Lebrin et al. 2004) and migration (Bautch 2017) of endothelial cells. More recent work has shown endoglin expression on a variety of cell (Schoonderwoerd et al. 2010). In our study, all cells tested were positive for ENG, although HAEC expression was very low (relative expression = 0.05). It shows that although endoglin is widely described as an endothelial marker, it is not a strong enough indicator. Moreover, the primary cell lines obtained from the inner layer have the highest gene expression compared to the control HAEC endothelial cells and other cell lines.

Intercellular adhesion molecule 2 (ICAM-2 belongs to the immunoglobulin (Ig) superfamily, constitutively expressed in endothelial junction (McLaughlin et al. 1998). ICAM-2 is concentrated at endothelial cell–cell contact (Huang et al. 2005). Several studies have shown that ICAM-2 expression on endothelial cells is concentrated at the junctions and is involved in leukocyte recruitment and angiogenesis, and is responsible for the regulation of endothelial barrier function and permeability (Huang et al. 2005; Amsellem et al. 2014). There are numerous reports of the use of ICAM2 as an endothelial surface marker (Gao et al. 2017; Sadek et al. 2008), which is upregulated during differentiation into mature vascular endothelial cells. ICAM-2 is also an activator of the PI3/AKT pathway leading to inhibition of apoptosis (Perez et al. 2002). Our flow cytometry analyses revealed that ICAM2 positive cells were the only control endothelial cells. Among the cells isolated from the internal layer, there were no ICAM2 positive cells. Lack of this marker indicated that there was a significant loss of endothelial cells in the internal layer of the aortic wall in comparison to control cells. Surprising was the fact that ICAM2 gene expression was the lowest in control endothelial cells HAEC in comparison to other cells (1038 and 1135 of fold change difference between the expression of HAEC in AoSMC and AoAF cells).

TEK (TIE2) is an angiopoietin receptor tyrosine kinase expressed in endothelial cells and their precursors. There is restriction in the distribution of TEK to the vascular endothelium (Sato et al. 1995; Dumont et al. 1994). TEK is inactivated during the process of angiogenesis and inflammation, which leads to neovascularization of the abdominal aortic aneurysm and increased infiltration of monocytes/macrophages (Yu et al. 2016).

Our study showed that TEK positive cells in control cell lines were observed only in HAEC. There was a lack of this marker in the cells from the external layer. There were some TEK positive cells in the internal and external layer of AAA. The highest expression of TEK gene was observed in AoSMC. There was no difference in expression in AoSMC and AAA mid-tier cell lines. Increased expression of TEK was observed in cell lines obtained from the inner layers of aneurysm compared to the standard HAEC line. Selection of a specific marker of cells isolated from layers of AAA could provide information about the mechanism of AAA formation. Unfortunately, none of the tested markers seems to be optimal and characteristic for a specific layer of AAA. This study showed that only ICAM2 was present only on the surface of HAEC cells. Its absence on the surface of cells from the inner AAA layer may indicate a change in the phenotype of these cells. Absence of ICAM2 on the surface of other control cells, as well as on the surface of cells from the middle and external layers would be desirable. However, this conflicts with the ICAM2 gene expression in all types of cells from all layers. CNN1, MYH10, and MYOCD were intended to be surface markers of SMC. At the mRNA level, there were differences between standard cell lines. Unfortunately, these differences did not translate into cell lines obtained from individual layers of the abdominal aortic aneurysm and proteins. Analysis of the expression of protein markers for specific cell lines is not enough. All proteins analyzed were intended to be cell-specific markers. Current molecular techniques for mRNA or protein analysis are very sensitive. Cells are living organisms that produce all kinds of mRNA and proteins in small amounts. The increasing sensitivity of molecular techniques makes the analysis cumbersome as increasing data can be obtained. The time lag between mRNA and protein expression may also explain the observed differences between expression analysis and flow cytometry.

## Supplementary Information

Below is the link to the electronic supplementary material.

Supplementary file1 (DOCX 19 KB)

Supplementary file2 (DOCX 22 KB)

Supplementary file3 (DOCX 28 KB)

Supplementary file4 (DOCX 30 KB)

Supplementary file5 (DOCX 38 KB)

## Data Availability

All data generated or analyzed during this study are included in this published article.
