# Supplementary material for: Searching for new molecular markers for cells obtained from abdominal aortic aneurysm
Source: J Appl Genet. 2021 Jun 2;62(3):487–97. doi: 10.1007/s13353-021-00641-4 (PMC8357660; doi:10.1007/s13353-021-00641-4)
Supplement: Supplementary file 1 — Supplementary file1 (DOCX 19 KB) [file 13353_2021_641_MOESM1_ESM.docx]

Supplement Table 1.

Supplement Table 1. Alphabetical list of genes used in the study with appropriate Assay ID (ROCHE), HGNC symbols and gene belonging to the marker family.

Three reference genes are listed at the beginning of the table.

| **Assay ID** | **Gene Symbol** | **Description** | **Marker family** |
| --- | --- | --- | --- |
| 141139 | *GAPDH H. sapiens* | glyceraldehyde-3-phosphate dehydrogenase [Source:HGNC Symbol;Acc:4141] | Reference gene |
| 102088 | *PPIA H. sapiens* | peptidylprolyl isomerase A (cyclophilin A) [Source:HGNC Symbol;Acc:9253] | Reference gene |
| 101144 | *RPL0 H. sapiens* | ribosomal protein, large, [Source:HGNC Symbol 10371] | Reference gene |
| 116657 | *ACTA1 H. sapiens* | actin, alpha 1, skeletal muscle [Source:HGNC Symbol Acc:129] | Smooth Muscle |
| 117143 | *ALCAM H. sapiens* | activated leukocyte cell adhesion molecule [Source:HGNC Symbol Acc:400] | Fibroblast (Stromal) Surface Markers; Activated T-cells |
| 140015 | *ANGPTL4 H. sapiens* | angiopoietin-like 4 [Source:HGNC Symbol Acc:16039] | Primary Endothelial Cells |
| 103025 | *CDH5 H. sapiens* | cadherin 5, type 2 (vascular endothelium) [Source:HGNC Symbol Acc:1764] | Endothelial |
| 111687 | *CNN1 H. sapiens* | calponin 1, basic, smooth muscle [Source:HGNC Symbol Acc:2155] | Smooth Muscle |
| 141032 | *CD163 H. sapiens* | CD163 molecule [Source:HGNC Symbol Acc:1631] | Other Monocyte and Macrophage Surface Markers |
| 115312 | *CD1A H. sapiens* | CD1a molecule [Source:HGNC Symbol Acc:1634] | Dendritic Cell Surface Markers |
| 149400 | *CD1D H. sapiens* | CD1d molecule [Source:HGNC Symbol Acc:1637] | Epithelial Cell Surface Markers |
| 113245 | *CD209 H. sapiens* | CD209 molecule [Source:HGNC Symbol Acc:1641] | Dendritic Cell Surface Markers |
| 146453 | *CD34 H. sapiens* | CD34 molecule [Source:HGNC Symbol Acc:1662] | Hematopoietic Stem/progenitor Cells, Vascular Endothelium and Some Fibroblasts |
| 100254 | *CD40 H. sapiens* | CD40 molecule, TNF receptor superfamily member 5 [Source:HGNC Symbol Acc:11919] | Other Monocyte and Macrophage Surface Markers; Dendritic Cell Surface Markers; Mature B-cells |
| 127686 | *CD68 H. sapiens* | CD68 molecule [Source:HGNC Symbol Acc:1693] | Other Monocyte and Macrophage Surface Markers |
| 146968 | *CD69 H. sapiens* | CD69 molecule [Source:HGNC Symbol Acc:1694] | Activated macrophages; Activated B-cells; Activated T-cells |
| 145747 | *CD70 H. sapiens* | CD70 molecule [Source:HGNC Symbol Acc:11937] | Other Monocyte and Macrophage Surface Markers; Activated B-cells; Activated T-cells |
| 115057 | *CD83 H. sapiens* | CD83 molecule [Source:HGNC Symbol Acc:1703] | Dendritic Cell Surface Markers; Activated B-cells; Activated T-cells |
| 113291 | *CD86 H. sapiens* | CD86 molecule [Source:HGNC Symbol Acc:1705] | Other Monocyte and Macrophage Surface Markers; Dendritic Cell Surface Markers; Activated B-cells |
| 105951 | *CSF1R H. sapiens* | colony stimulating factor 1 receptor [Source:HGNC Symbol Acc:2433] | Other Monocyte and Macrophage Surface Markers |
| 149385 | *C5AR1 H. sapiens* | complement component 5a receptor 1 [Source:HGNC Symbol Acc:1338] | Other Monocyte and Macrophage Surface Markers; Mast Cell Surface Markers |
| 148619 | *DDR2 H. sapiens* | discoidin domain receptor tyrosine kinase 2 [Source:HGNC Symbol Acc:2731] | Smooth Muscle |
| 104599 | *ENG H. sapiens* | endoglin [Source:HGNC Symbol Acc:3349] | Activated Macrophages; Endothelial Cell Surface Markers |
| 149391 | *EPCAM H. sapiens* | epithelial cell adhesion molecule [Source:HGNC Symbol Acc:11529] | Epithelial Cell Surface Markers |
| 137865 | *FCER2 H. sapiens* | Fc fragment of IgE, low affinity II, receptor for (CD23) [Source:HGNC Symbol Acc:3612] | Activated macrophages; Dendritic Cell Surface Markers; Mast Cell Surface Markers; Activated B-cells |
| 110762 | *ITGA1 H. sapiens* | integrin, alpha 1 [Source:HGNC Symbol Acc:6134] | Other Monocyte and Macrophage Surface Markers; Activated T-cells |
| 111263 | *ITGA2 H. sapiens* | integrin, alpha 2 (CD49B, alpha 2 subunit of VLA-2 receptor) [Source:HGNC Symbol Acc:6137] | Other Monocyte and Macrophage Surface Markers; Mature B-cells |
| 126792 | *ICAM2 H. sapiens* | intercellular adhesion molecule 2 [Source:HGNC Symbol Acc:5345] | Other Monocyte and Macrophage Surface Markers; Endothelial Cell Surface Markers |
| 144625 | *IL1R2 H. sapiens* | interleukin 1 receptor, type II [Source:HGNC Symbol Acc:5994] | Other Monocyte and Macrophage Surface Markers; Mature B-cells |
| 111304 | *IL2RA H. sapiens* | interleukin 2 receptor, alpha [Source:HGNC Symbol Acc:6008] | Activated macrophages; Activated B-cells; Activated T-cells |
| 113761 | *KRT5 H. sapiens* | keratin 5 [Source:HGNC Symbol Acc:6442] | Epithelial Cell Surface Markers |
| 114196 | *KRT8 H. sapiens* | keratin 8 [Source:HGNC Symbol Acc:6446] | Epithelial Cell Surface Markers |
| 137437 | *AC107016.2 H. sapiens* | Keratin, type I cytoskeletal 18 (Cytokeratin-18)(CK-18)(Keratin-18)(K18)(Cell proliferation-inducing gene 46 protein) [Source:UniProtKB/Swiss-Prot Acc:P05783] | Epithelial Cell Surface Markers; miRNA |
| 119171 | *MYOCD H. sapiens* | myocardin [Source:HGNC Symbol Acc:16067] | Smooth Muscle Cell Surface Markers |
| 116420 | *MYH10 H. sapiens* | myosin, heavy chain 10, non-muscle [Source:HGNC Symbol Acc:12726] | Smooth Muscle Cell Surface Markers |
| 148296 | *MYH9 H. sapiens* | myosin, heavy chain 9, non-muscle [Source:HGNC Symbol Acc:7568] | Smooth Muscle Cell Surface Markers |
| 139599 | *NOS3 H. sapiens* | nitric oxide synthase 3 (endothelial cell) [Source:HGNC Symbol Acc:7876] | Endothelial Cell Surface Markers |
| 148187 | *PECAM1 H. sapiens* | platelet/endothelial cell adhesion molecule [Source:HGNC Symbol Acc:8823] | Endothelial Cell Surface Markers |
| 107833 | *RETN H. sapiens* | resistin [Source:HGNC Symbol Acc:20389] | Adipocyte Surface Markers |
| 110779 | *S100A4 H. sapiens* | S100 calcium binding protein A4 [Source:HGNC Symbol Acc:10494] | Fibroblast (Stromal) Surface Markers |
| 144226 | *S100A8 H. sapiens* | S100 calcium binding protein A8 [Source:HGNC Symbol Acc:10498] | Other Monocyte and Macrophage Surface Markers |
| 113067 | *SELP H. sapiens* | selectin P (granule membrane protein 140kDa, antigen CD62) [Source:HGNC Symbol Acc:10721] | Endothelial Cell Surface Markers |
| 138461 | *SMTN H. sapiens* | smoothelin [Source:HGNC Symbol Acc:11126] | Smooth Muscle |
| 105772 | *TEK H. sapiens* | TEK tyrosine kinase, endothelial [Source:HGNC Symbol Acc:11724] | Endothelial Cell Surface Markers |
| 116810 | *THY1 H. sapiens* | Thy-1 cell surface antigen [Source:HGNC Symbol Acc:11801] | Fibroblast; mature neurons, a subset of fibroblasts, and activated natural killer cells |
| 145468 | *TNFRSF8 H. sapiens* | tumor necrosis factor receptor superfamily, member 8 [Source:HGNC Symbol Acc:11923] | Other Monocyte and Macrophage Surface Markers; Activated B-cells; Activated T-cells |
| 103286 | *VCAM1 H. sapiens* | vascular cell adhesion molecule 1 [Source:HGNC Symbol Acc:12663] | Endothelial Cell Surface Markers |
| 103244 | *VWF H. sapiens* | von Willebrand factor [Source:HGNC Symbol Acc:12726] | Endothelial Cell Surface Markers |
