# Supplementary material for: Searching for new molecular markers for cells obtained from abdominal aortic aneurysm
Source: J Appl Genet. 2021 Jun 2;62(3):487–97. doi: 10.1007/s13353-021-00641-4 (PMC8357660; doi:10.1007/s13353-021-00641-4)
Supplement: Supplementary file 2 — Supplementary file2 (DOCX 22 KB) [file 13353_2021_641_MOESM2_ESM.docx]

**Supplement Table 2. P-value for T-test, 1-tailed.** Statistical significance analysis for the expression analysis between cells derived from different layers of AAA.

| **GENES**  **Cells vs cells** | ***ALCAM*** | ***ANGPTL4*** | ***CD40*** | ***CNN1*** | ***EPCAM*** | ***ITGA1*** | ***ITGA2*** | ***KRT18/***  ***AC107016,2*** | ***KRT8*** | ***MYH9*** | ***VCAM1*** |
| --- | --- | --- | --- | --- | --- | --- | --- | --- | --- | --- | --- |
| **AoSMC vs HAEC** | 0,040227 | 0,167364 | 1,00E-08 | 7,31E-04 | 3,90E-07 | 3,28E-05 | 0,029122 | 0,010821 | 7,79E-04 | 0,005347 | 0,341471 |
| **AoSMC vs AoAF** | 8,41E-04 | 1,00E-08 | 5,10E-07 | 1,87E-05 | 0,063163 | 0,211406 | 0,228547 | 0,00152 | 1,00E-08 | 0,210402 | 4,50E-05 |
| **HAEC vs AoAF** | 1,69E-05 | 6,78E-06 | 1,00E-08 | 0,001169794 | 0,312944 | 4,62E-06 | 0,001191 | 0,018615 | 2,94E-06 | 2,39E-05 | 2,64E-05 |
| **AoSMC vs ML1** | 0,110566 | 9,90E-05 | 0,491126 | 1,34E-07 | 0,004124 | 0,077447 | 0,45275 | 0,077607 | 0,479828 | 0,147077 | 0,320675 |
| **AoSMC vs ML2** | 0,422753 | 6,79E-06 | 0,47439 | 2,59E-06 | 0,000405 | 0,174401 | 0,324081 | 0,478956 | 0,001232 | 0,000448 | 0,112683 |
| **AoSMC vs ML3** | 0,27888 | 0,020607 | 0,030991 | 2,09E-05 | 0,198497 | 0,353626 | 0,449763 | 0,023436 | 0,013854 | 0,003356 | 0,207496 |
| **ML1 vs ML2** | 0,073217 | 0,002788 | 0,47402 | 0,260238571 | 0,337006 | 0,304051 | 0,342585 | 0,0495 | 0,00467 | 0,005386 | 0,29827 |
| **ML1 vs ML3** | 0,034557 | 0,456801 | 0,039746 | 0,403532317 | 0,262132 | 0,078948 | 0,376182 | 0,303196 | 0,016291 | 0,01994 | 0,135628 |
| **ML2 vs ML3** | 0,329174 | 0,094368 | 0,035558 | 0,394877738 | 0,186259 | 0,144584 | 0,242217 | 0,012264 | 0,229153 | 0,414703 | 0,053159 |
| **HAEC vs IL1** | 0,332301 | 6,68E-05 | 0,00016 | 0,410552939 | 0,299721 | 0,001508 | 6,72E-05 | 3,50E-05 | 0,005918 | 2,12E-07 | 0,457627 |
| **HAEC vs IL2** | 0,208578 | 0,006278 | 3,12E-07 | 0,347933702 | 0,398553 | 0,015098 | 0,002451 | 0,001822 | 0,008029 | 1,84E-06 | 0,327417 |
| **HAEC vs IL3** | 0,093834 | 0,045301 | 3,20E-08 | 0,275847034 | 0,042534 | 0,094063 | 0,000251 | 0,00825 | 0,142298 | 2,88E-07 | 0,251096 |
| **IL1 vs IL2** | 0,120321 | 0,088314 | 0,079603 | 0,133925708 | 0,280677 | 0,158017 | 0,253332 | 0,1022 | 0,197527 | 0,130844 | 0,294764 |
| **IL1 vs IL3** | 0,053005 | 0,068659 | 0,243698 | 0,274053015 | 0,10825 | 0,015515 | 0,060912 | 0,106434 | 0,202154 | 0,013863 | 0,221584 |
| **IL2 vs IL3** | 0,211584 | 0,330596 | 0,121987 | 0,144665253 | 0,059656 | 0,125546 | 0,267893 | 0,36723 | 0,079905 | 0,216033 | 0,412884 |
| **AoAF vs EL1** | 0,000318 | 4,10E-08 | 0,007761 | 0,290071516 | 0,196683 | 1,89E-05 | 0,001772 | 0,193017 | 1,00E-08 | 2,67E-05 | 5,07E-05 |
| **AoAF vs EL2** | 0,278661 | 1,00E-08 | 0,000767 | 0,086413988 | 0,184104 | 0,055285 | 0,148579 | 0,005704 | 0,000932 | 0,027485 | 0,385334 |
| **AoAF vs EL3** | 0,02231 | 8,40E-08 | 0,000216 | 0,012000124 | 0,104717 | 0,158012 | 0,377977 | 0,00037 | 7,10E-07 | 0,013675 | 0,039874 |
| **EL1 vs EL2** | 0,072432 | 2,23E-05 | 0,1001 | 0,402519664 | 0,434625 | 4,13E-05 | 0,092139 | 0,069976 | 0,02366 | 0,002451 | 0,046784 |
| **EL1 vs EL3** | 0,001076 | 0,00015 | 0,056064 | 0,049754567 | 0,134205 | 0,12352 | 0,008562 | 0,003144 | 2,50E-08 | 4,42E-05 | 0,053892 |
| **EL2 vs EL3** | 0,002684 | 0,005649 | 0,008112 | 0,026365128 | 0,02721 | 0,048881 | 0,098551 | 0,153867 | 0,161776 | 0,384692 | 0,414577 |
