# Supplementary material for: Searching for new molecular markers for cells obtained from abdominal aortic aneurysm
Source: J Appl Genet. 2021 Jun 2;62(3):487–97. doi: 10.1007/s13353-021-00641-4 (PMC8357660; doi:10.1007/s13353-021-00641-4)
Supplement: Supplementary file 3 — Supplementary file3 (DOCX 28 KB) [file 13353_2021_641_MOESM3_ESM.docx]

**Supplement Table 3. P-value for Mann-Whitney test, 1-tailed.** Analysis of statistical significance for the difference in expression between cells derived from different layers of AAA.

| **Cells vs cells**  **GENES** | **AoSMC vs HAEC** | **AoSMC vs AoAF** | **HAEC vs AoAF** | **AoSMC vs ML1** | **AoSMC vs ML2** | **AoSMC vs ML3** | **ML1 vs ML2** | **ML1 vs ML3** | **ML2 vs ML3** | **HAEC vs IL1** | **HAEC vs IL2** | **HAEC vs IL3** | **IL1 vs IL2** | **IL1 vs IL3** | **IL2 vs IL3** | **AoAF vs EL1** | **AoAF vs EL2** | **AoAF vs EL3** | **EL1 vs EL2** | **EL1 vs EL3** | **EL2 vs EL3** |
| --- | --- | --- | --- | --- | --- | --- | --- | --- | --- | --- | --- | --- | --- | --- | --- | --- | --- | --- | --- | --- | --- |
| ***ACTA1*** | 0,212 | 0,472 | 0,112 | 0,344 | 0,500 | 0,334 | 0,344 | 0,177 | 0,334 | 0,112 | 0,098 | 0,077 | 0,500 | 0,351 | 0,364 | 0,360 | 0,351 | 0,305 | 0,472 | 0,216 | 0,203 |
| ***C5AR1*** | 0,087 | 0,002 | 0,002 | 0,003 | 0,064 | 0,009 | 0,405 | 0,309 | 0,260 | 0,006 | 0,031 | 0,472 | 0,177 | 0,186 | 0,021 | 0,002 | 0,005 | 0,001 | 0,127 | 0,004 | 0,327 |
| ***CD163*** | 0,344 | 0,472 | 0,360 | 0,033 | 0,261 | 0,032 | 0,236 | 0,216 | 0,284 | 0,002 | 0,004 | 0,019 | 0,364 | 0,101 | 0,262 | 0,472 | 0,500 | 0,500 | 0,472 | 0,472 | 0,500 |
| ***CD1A*** | 0,344 | 0,360 | 0,472 | 0,344 | 0,344 | 0,112 | 0,500 | 0,309 | 0,309 | 0,472 | 0,373 | 0,334 | 0,343 | 0,351 | 0,226 | 0,360 | 0,222 | 0,351 | 0,112 | 0,472 | 0,101 |
| ***CD1D*** | 0,344 | 0,002 | 0,002 | 0,288 | 0,405 | 0,142 | 0,405 | 0,260 | 0,216 | 0,027 | 0,474 | 0,360 | 0,149 | 0,125 | 0,301 | 0,002 | 0,015 | 0,001 | 0,019 | 0,019 | 0,015 |
| ***CD209*** | 0,500 | 0,334 | 0,334 | 0,344 | 0,405 | 0,309 | 0,189 | 0,112 | 0,472 | 0,472 | 0,500 | 0,472 | 0,477 | 0,500 | 0,477 | 0,472 | 0,351 | 0,475 | 0,360 | 0,472 | 0,351 |
| ***CD34*** | 0,003 | 0,027 | 0,002 | 0,236 | 0,115 | 0,037 | 0,405 | 0,112 | 0,112 | 0,002 | 0,001 | 0,002 | 0,364 | 0,305 | 0,226 | 0,027 | 0,399 | 0,001 | 0,019 | 0,388 | 0,001 |
| ***CD68*** | 0,003 | 0,472 | 0,002 | 0,115 | 0,064 | 0,360 | 0,003 | 0,023 | 0,177 | 0,002 | 0,001 | 0,002 | 0,032 | 0,399 | 0,092 | 0,067 | 0,002 | 0,020 | 0,472 | 0,260 | 0,399 |
| ***CD69*** | 0,288 | 0,002 | 0,002 | 0,189 | 0,087 | 0,037 | 0,405 | 0,415 | 0,360 | 0,004 | 0,017 | 0,360 | 0,322 | 0,080 | 0,028 | 0,002 | 0,005 | 0,001 | 0,006 | 0,027 | 0,048 |
| ***CD70*** | 0,004 | 0,002 | 0,002 | 0,003 | 0,003 | 0,309 | 0,405 | 0,216 | 0,260 | 0,006 | 0,031 | 0,360 | 0,082 | 0,305 | 0,074 | 0,002 | 0,125 | 0,001 | 0,006 | 0,472 | 0,005 |
| ***CD83*** | 0,003 | 0,360 | 0,002 | 0,468 | 0,023 | 0,112 | 0,149 | 0,087 | 0,472 | 0,013 | 0,041 | 0,177 | 0,322 | 0,153 | 0,343 | 0,019 | 0,261 | 0,261 | 0,177 | 0,177 | 0,305 |
| ***CD86*** | 0,468 | 0,216 | 0,216 | 0,189 | 0,344 | 0,050 | 0,405 | 0,415 | 0,196 | 0,472 | 0,239 | 0,112 | 0,226 | 0,101 | 0,386 | 0,334 | 0,475 | 0,203 | 0,334 | 0,334 | 0,203 |
| ***CD90/***  ***THY1*** | 0,003 | 0,027 | 0,002 | 0,189 | 0,315 | 0,037 | 0,087 | 0,472 | 0,019 | 0,002 | 0,001 | 0,002 | 0,149 | 0,399 | 0,343 | 0,002 | 0,020 | 0,001 | 0,415 | 0,002 | 0,101 |
| ***CDH5*** | 0,003 | 0,003 | 0,002 | 0,033 | 0,189 | 0,360 | 0,288 | 0,013 | 0,472 | 0,360 | 0,239 | 0,360 | 0,124 | 0,449 | 0,136 | 0,177 | 0,001 | 0,015 | 0,027 | 0,050 | 0,351 |
| ***CSF1R*** | 0,033 | 0,216 | 0,002 | 0,344 | 0,149 | 0,112 | 0,087 | 0,050 | 0,216 | 0,019 | 0,001 | 0,050 | 0,177 | 0,305 | 0,092 | 0,177 | 0,500 | 0,351 | 0,099 | 0,216 | 0,449 |
| ***DDR2*** | 0,003 | 0,415 | 0,002 | 0,189 | 0,064 | 0,050 | 0,288 | 0,112 | 0,360 | 0,002 | 0,001 | 0,013 | 0,066 | 0,020 | 0,343 | 0,142 | 0,186 | 0,261 | 0,067 | 0,284 | 0,153 |
| ***ENG*** | 0,003 | 0,043 | 0,002 | 0,344 | 0,033 | 0,142 | 0,007 | 0,142 | 0,002 | 0,002 | 0,001 | 0,002 | 0,053 | 0,351 | 0,343 | 0,360 | 0,001 | 0,500 | 0,050 | 0,260 | 0,015 |
| ***FCER2*** | 0,149 | 0,112 | 0,112 | 0,003 | 0,007 | 0,037 | 0,405 | 0,177 | 0,177 | 0,003 | 0,023 | 0,360 | 0,500 | 0,048 | 0,066 | 0,112 | 0,449 | 0,101 | 0,037 | 0,360 | 0,015 |
| ***ICAM2*** | 0,003 | 0,500 | 0,002 | 0,033 | 0,003 | 0,009 | 0,023 | 0,037 | 0,177 | 0,002 | 0,001 | 0,002 | 0,408 | 0,351 | 0,021 | 0,002 | 0,048 | 0,005 | 0,050 | 0,112 | 0,125 |
| ***IL1R2*** | 0,003 | 0,037 | 0,002 | 0,010 | 0,023 | 0,019 | 0,236 | 0,142 | 0,472 | 0,002 | 0,001 | 0,002 | 0,047 | 0,500 | 0,092 | 0,472 | 0,015 | 0,028 | 0,196 | 0,415 | 0,063 |
| ***IL2RA*** | 0,468 | 0,472 | 0,472 | 0,344 | 0,344 | 0,112 | 0,500 | 0,309 | 0,309 | 0,360 | 0,373 | 0,360 | 0,477 | 0,500 | 0,477 | 0,472 | 0,475 | 0,475 | 0,472 | 0,472 | 0,475 |
| ***KRT5*** | 0,500 | 0,472 | 0,472 | 0,500 | 0,288 | 0,334 | 0,405 | 0,334 | 0,177 | 0,099 | 0,423 | 0,388 | 0,112 | 0,203 | 0,431 | 0,216 | 0,261 | 0,399 | 0,415 | 0,159 | 0,305 |
| ***MYH10*** | 0,003 | 0,027 | 0,002 | 0,064 | 0,003 | 0,002 | 0,015 | 0,037 | 0,360 | 0,002 | 0,001 | 0,002 | 0,053 | 0,011 | 0,163 | 0,003 | 0,475 | 0,002 | 0,360 | 0,002 | 0,005 |
| ***MYOCD*** | 0,003 | 0,002 | 0,002 | 0,003 | 0,003 | 0,002 | 0,087 | 0,216 | 0,112 | 0,019 | 0,012 | 0,360 | 0,364 | 0,080 | 0,047 | 0,002 | 0,004 | 0,261 | 0,360 | 0,360 | 0,080 |
| ***NOS3*** | 0,003 | 0,472 | 0,002 | 0,344 | 0,189 | 0,360 | 0,344 | 0,472 | 0,360 | 0,019 | 0,001 | 0,019 | 0,343 | 0,500 | 0,343 | 0,472 | 0,475 | 0,475 | 0,472 | 0,472 | 0,475 |
| ***PECAM1*** | 0,003 | 0,002 | 0,002 | 0,003 | 0,007 | 0,002 | 0,468 | 0,216 | 0,260 | 0,002 | 0,001 | 0,002 | 0,082 | 0,449 | 0,262 | 0,002 | 0,011 | 0,001 | 0,003 | 0,472 | 0,002 |
| ***RETN*** | 0,003 | 0,112 | 0,112 | 0,189 | 0,003 | 0,002 | 0,064 | 0,019 | 0,067 | 0,003 | 0,303 | 0,360 | 0,244 | 0,063 | 0,343 | 0,112 | 0,449 | 0,101 | 0,004 | 0,472 | 0,005 |
| ***S100A4*** | 0,003 | 0,002 | 0,002 | 0,003 | 0,003 | 0,002 | 0,064 | 0,009 | 0,142 | 0,002 | 0,001 | 0,002 | 0,322 | 0,261 | 0,193 | 0,260 | 0,002 | 0,001 | 0,067 | 0,177 | 0,186 |
| ***S100A8*** | 0,023 | 0,415 | 0,472 | 0,004 | 0,003 | 0,112 | 0,405 | 0,360 | 0,415 | 0,260 | 0,239 | 0,260 | 0,124 | 0,080 | 0,262 | 0,472 | 0,424 | 0,500 | 0,019 | 0,472 | 0,015 |
| ***SELP*** | 0,468 | 0,334 | 0,472 | 0,087 | 0,236 | 0,112 | 0,189 | 0,360 | 0,309 | 0,002 | 0,001 | 0,002 | 0,477 | 0,475 | 0,477 | 0,196 | 0,153 | 0,186 | 0,472 | 0,087 | 0,063 |
| ***SMTN*** | 0,003 | 0,177 | 0,002 | 0,115 | 0,033 | 0,216 | 0,344 | 0,260 | 0,159 | 0,009 | 0,326 | 0,009 | 0,053 | 0,222 | 0,016 | 0,008 | 0,004 | 0,015 | 0,023 | 0,309 | 0,222 |
| ***TEK*** | 0,003 | 0,002 | 0,002 | 0,405 | 0,149 | 0,177 | 0,468 | 0,216 | 0,177 | 0,002 | 0,001 | 0,112 | 0,408 | 0,449 | 0,301 | 0,002 | 0,001 | 0,020 | 0,112 | 0,360 | 0,080 |
| ***TNFRSF8*** | 0,189 | 0,004 | 0,177 | 0,189 | 0,149 | 0,472 | 0,087 | 0,472 | 0,415 | 0,177 | 0,166 | 0,472 | 0,454 | 0,048 | 0,092 | 0,360 | 0,153 | 0,399 | 0,260 | 0,388 | 0,305 |
| ***VWF*** | 0,003 | 0,050 | 0,002 | 0,003 | 0,003 | 0,002 | 0,033 | 0,067 | 0,309 | 0,002 | 0,001 | 0,002 | 0,408 | 0,037 | 0,008 | 0,027 | 0,015 | 0,015 | 0,415 | 0,142 | 0,222 |
