# Supplementary material for: Searching for new molecular markers for cells obtained from abdominal aortic aneurysm
Source: J Appl Genet. 2021 Jun 2;62(3):487–97. doi: 10.1007/s13353-021-00641-4 (PMC8357660; doi:10.1007/s13353-021-00641-4)
Supplement: Supplementary file 4 — Supplementary file4 (DOCX 30 KB) [file 13353_2021_641_MOESM4_ESM.docx]

**Supplement Table 4. Fold change differences between cells derived from different layers of AAA.**

| **Cells vs cells**  **GENES** | **AoSMC vs HAEC** | **AoSMC vs AoAF** | **HAEC vs AoAF** | **AoSMC vs ML1** | **AoSMC vs ML2** | **AoSMC vs ML3** | **ML1 vs ML2** | **ML1 vs ML3** | **ML2 vs ML3** | **HAEC vs IL1** | **HAEC vs IL2** | **HAEC vs IL3** | **IL1 vs IL2** | **IL1 vs IL3** | **IL2 vs IL3** | **AoAF vs EL1** | **AoAF vs EL2** | **AoAF vs EL3** | **EL1 vs EL2** | **EL1 vs EL3** | **EL2 vs EL3** |
| --- | --- | --- | --- | --- | --- | --- | --- | --- | --- | --- | --- | --- | --- | --- | --- | --- | --- | --- | --- | --- | --- |
| ***ACTA1*** | -43 | 1 | 61 | -35 | -1 | 6 | 34 | 220 | 6 | 65 | 78 | 271 | 1 | 4 | 3 | 4 | 4 | -16 | 1 | -71 | -71 |
| ***ALCAM*** | -2 | 2 | 3 | 1 | -1 | -1 | -1 | -2 | -1 | 1 | -1 | -2 | -1 | -2 | -1 | 1 | 1 | -1 | -1 | -2 | -2 |
| ***ANGPTL4*** | 2 | -12 | -20 | 3 | 5 | 3 | 2 | 1 | -2 | 17 | 7 | 5 | -2 | -3 | -1 | 8 | 108 | 92 | 14 | 12 | -1 |
| ***C5AR1*** | -1 | -8 | -6 | -3 | -4 | -8 | -1 | -2 | -2 | -3 | -7 | 1 | -2 | 5 | 11 | 11 | 5 | 8 | -2 | -1 | 2 |
| ***CD163*** | 11 | 1 | -9 | -13682 | -59 | -3143 | 231 | 4 | -53 | -273818 | -26181 | -4096 | 10 | 67 | 6 | 1 | 2 | 2 | 1 | 1 | 1 |
| ***CD1A*** | -29 | -6 | 5 | -8 | -31 | -92 | -4 | -12 | -3 | 5 | -10 | 29 | -50 | 5 | 277 | 6 | -47 | 6 | -297 | 1 | 297 |
| ***CD1D*** | 1 | -36 | -43 | -1 | -1 | -2 | 1 | -1 | -2 | -2 | -1 | -1 | 1 | 2 | 1 | 49 | 21 | 24 | -2 | -2 | 1 |
| ***CD209*** | -1 | 11 | 15 | 11 | -3 | -4 | -34 | -45 | -1 | 3 | 2 | 1 | -2 | -2 | -1 | 1 | -6 | 1 | -6 | 1 | 6 |
| ***CD34*** | 3405 | 3 | -1277 | 5 | 4 | 2 | -1 | -2 | -2 | -896 | -1779 | -715 | -2 | 1 | 2 | 8 | -1 | 10 | -8 | 1 | 10 |
| ***CD40*** | 18 | -12 | -215 | -1 | 1 | 4 | 1 | 4 | 4 | -4 | -6 | -5 | -2 | -1 | 1 | 3 | 5 | 6 | 2 | 2 | 1 |
| ***CD68*** | -5 | 1 | 6 | -1 | 1 | 1 | 2 | 1 | -1 | 7 | 6 | 7 | -1 | 1 | 1 | -2 | -2 | -2 | -1 | -1 | -1 |
| ***CD69*** | 1 | -43 | -51 | -3 | -2 | -2 | 1 | 1 | 1 | -4 | -8 | -1 | -2 | 3 | 6 | 73 | 14 | 38 | -5 | -2 | 3 |
| ***CD70*** | -1 | -13 | -9 | -10 | -6 | -5 | 2 | 2 | 1 | -3 | -8 | -2 | -3 | 1 | 3 | 7 | -1 | 7 | -9 | -1 | 9 |
| ***CD83*** | 9 | 1 | -6 | 1 | 3 | 9 | 3 | 9 | 3 | 2 | 2 | 1 | -1 | -2 | -1 | -2 | -1 | -1 | 2 | 2 | 1 |
| ***CD86*** | 1 | -23 | -23 | -33 | -7 | -73 | 5 | -2 | -10 | 1 | -18 | -103 | -18 | -103 | -6 | 4 | -1 | 23 | -5 | 5 | 25 |
| ***CD90/THY1*** | -48 | 1 | 59 | 1 | -1 | 1 | -1 | 1 | 1 | 22 | 29 | 24 | 1 | 1 | -1 | 3 | 2 | 2 | -1 | -2 | -1 |
| ***CDH5*** | 629 | 3 | -244 | -13 | 16 | 14 | 203 | 178 | -1 | -44 | -653 | -53 | -15 | -1 | 12 | -4 | 365 | 86 | 1635 | 387 | -4 |
| ***CNN1*** | -74 | -2 | 36 | -8 | -10 | -9 | -1 | -1 | 1 | 1 | -1 | 3 | -2 | 2 | 4 | 1 | 1 | -2 | 1 | -3 | -3 |
| ***CSF1R*** | 5846 | -18 | -106183 | -23 | -2 | 3 | 11 | 75 | 7 | -4567 | -125587 | -880 | -27 | 5 | 143 | 9 | 3 | 15 | -3 | 2 | 4 |
| ***DDR2*** | -5 | 1 | 5 | -1 | -1 | -1 | -1 | -1 | -1 | 7 | 4 | 3 | -2 | -2 | -1 | 1 | -1 | 1 | -1 | -1 | 1 |
| ***ENG*** | 3 | 1 | -2 | 1 | 2 | -1 | 1 | -1 | -2 | -3 | -4 | -5 | -1 | -2 | -1 | -1 | -2 | -1 | -1 | 1 | 2 |
| ***EPCAM*** | -6 | -13 | -2 | -9 | -13 | -3 | -1 | 3 | 4 | 1 | -1 | 3 | -1 | 2 | 3 | 4 | 4 | 7 | -1 | 2 | 2 |
| ***FCER2*** | -1 | -3 | -3 | -60 | -8 | -5 | 7 | 12 | 2 | -7 | -7 | 1 | 1 | 10 | 9 | 2 | -7 | 2 | -13 | 1 | 14 |
| ***ICAM2*** | 1038 | -1 | -1136 | 2 | 5 | 8 | 3 | 4 | 2 | -135 | -142 | -112 | -1 | 1 | 1 | 236 | 2 | 3 | -138 | -93 | 1 |
| ***IL1R2*** | 1920 | -2 | -4646 | -7 | -4 | -4 | 2 | 2 | 1 | -803 | -1303 | -886 | -2 | -1 | 1 | 33 | -1 | -2 | -33 | -56 | -2 |
| ***IL2RA*** | 1 | 1 | 1 | -6 | -6 | -22 | -1 | -4 | -4 | -5 | -4 | -5 | 1 | 1 | -1 | 1 | 1 | 1 | 1 | 1 | 1 |
| ***ITGA1*** | -7 | -1 | 6 | 1 | 1 | -1 | -1 | -2 | -1 | 3 | 2 | 2 | -1 | -2 | -1 | 2 | -1 | 1 | -3 | -1 | 2 |
| ***ITGA2*** | 2 | 1 | -2 | -1 | -1 | 1 | -1 | 1 | 1 | -4 | -5 | -7 | -1 | -2 | -1 | 2 | 1 | -1 | -2 | -2 | -1 |
| ***KRT18/***  ***AC107016,2*** | -3 | -5 | -2 | -3 | 1 | -5 | 3 | -2 | -5 | -2 | -4 | -5 | -2 | -2 | -1 | 1 | 2 | 3 | 1 | 3 | 2 |
| ***KRT5*** | -1 | -2 | -1 | -3 | -56 | 15 | -20 | 41 | 821 | -2570 | -3 | -11 | 847 | 227 | -4 | -397 | -84 | -6 | 5 | 71 | 15 |
| ***KRT8*** | -2 | -5 | -3 | 1 | -2 | -4 | -2 | -4 | -2 | -3 | -4 | -2 | -2 | 2 | 2 | 15 | 5 | 3 | -3 | -5 | -2 |
| ***MYH10*** | -4 | -2 | 2 | -2 | -6 | -6 | -2 | -3 | -1 | -4 | -6 | -10 | -2 | -3 | -2 | 1 | 1 | -2 | -1 | -3 | -3 |
| ***MYH9*** | 2 | -1 | -2 | -1 | -3 | -3 | -2 | -2 | -1 | -6 | -8 | -10 | -1 | -2 | -1 | 2 | 1 | -1 | -2 | -3 | -2 |
| ***MYOCD*** | 94 | -47 | -4444 | -15 | -23 | -16 | -2 | -1 | 1 | -1250 | -2368 | -5 | -2 | 245 | 463 | 4 | 5 | 2 | 1 | -2 | -2 |
| ***NOS3*** | -51 | 1 | 51 | -5 | -22 | -4 | -5 | 1 | 5 | 14 | 51 | 13 | 4 | -1 | -4 | 1 | 1 | 1 | 1 | 1 | 1 |
| ***PECAM1*** | 197 | -29 | -5627 | -5 | -6 | -10 | -1 | -2 | -2 | -850 | -1920 | -1225 | -2 | -1 | 2 | 18 | 4 | 17 | -4 | -1 | 4 |
| ***RETN*** | -2 | -6 | -4 | 2 | -11 | -62 | -24 | -130 | -5 | -7 | -1 | 1 | 5 | 7 | 2 | 3 | -4 | 3 | -11 | 1 | 11 |
| ***S100A4*** | -21 | 4 | 89 | 18 | 11 | 9 | -2 | -2 | -1 | 185 | 227 | 405 | 1 | 2 | 2 | 2 | 4 | 2 | 2 | 1 | -2 |
| ***S100A8*** | -2 | 32 | 62 | -13 | -15 | -1 | -1 | 10 | 12 | 31 | 2 | -3 | -13 | -108 | -8 | -79 | -55 | -74 | 1 | 1 | -1 |
| ***SELP*** | -20 | -1 | 16 | 305 | 8 | 66 | -37 | -5 | 8 | 6173 | 6173 | 6173 | 1 | 1 | 1 | 57 | 71 | -7 | 1 | -402 | -498 |
| ***SMTN*** | -163 | 11 | 1732 | -13 | -48 | -5 | -4 | 2 | 9 | 82 | 5 | 594 | -17 | 7 | 122 | -231 | -492 | -146 | -2 | 2 | 3 |
| ***TEK*** | 8 | 2 | -4 | -1 | 1 | 1 | 1 | 1 | -1 | -38 | -54 | -12 | -1 | 3 | 5 | -5 | -12 | -4 | -3 | 1 | 3 |
| ***TNFRSF8*** | 30 | -5 | -151 | -2 | 1 | 2 | 3 | 5 | 2 | -84 | -72 | -2 | 1 | 52 | 44 | 8 | 2 | 5 | -4 | -2 | 2 |
| ***VCAM1*** | -1 | -9 | -7 | -1 | -2 | 2 | -1 | 3 | 4 | 1 | -1 | -2 | -1 | -2 | -1 | 4 | -1 | 2 | -5 | -2 | 2 |
| ***VWF*** | 42840 | 6 | -6995 | 6 | 4 | 4 | -1 | -1 | 1 | -4137 | -4417 | -2523 | -1 | 2 | 2 | -1 | 1 | 1 | 1 | 1 | 1 |
