# Supplementary material for: Searching for new molecular markers for cells obtained from abdominal aortic aneurysm
Source: J Appl Genet. 2021 Jun 2;62(3):487–97. doi: 10.1007/s13353-021-00641-4 (PMC8357660; doi:10.1007/s13353-021-00641-4)
Supplement: Supplementary file 5 — Supplementary file5 (DOCX 38 KB) [file 13353_2021_641_MOESM5_ESM.docx]

**Supplement Table 5. Relative expression of analyzed genes with standard error.**

| **Relative expression & SD**  **Gene** | **HAEC** | | **IL1** | | **IL2** | | **IL3** | | **AOSMC** | | **ML1** | | **ML2** | | **ML3** | | **AOAF** | | **EL1** | | **EL2** | | **EL3** | |
| --- | --- | --- | --- | --- | --- | --- | --- | --- | --- | --- | --- | --- | --- | --- | --- | --- | --- | --- | --- | --- | --- | --- | --- | --- |
|  | **ΔΔC_t_** | **SD** | **ΔΔC_t_** | **SD** | **ΔΔC_t_** | **SD** | **ΔΔC_t_** | **SD** | **ΔΔC_t_** | **SD** | **ΔΔC_t_** | **SD** | **ΔΔC_t_** | **SD** | **ΔΔC_t_** | **SD** | **ΔΔC_t_** | **SD** | **ΔΔC_t_** | **SD** | **ΔΔC_t_** | **SD** | **ΔΔC_t_** | **SD** |
| ***ACTA1*** | 8,1 | 8,9 | 2,1 | 5,4 | 1,8 | 5,1 | 0,0 | 0,0 | 2,7 | 6,5 | 7,8 | 13,0 | 2,7 | 6,6 | 0,0 | 0,0 | 2,1 | 5,7 | 0,0 | 0,0 | 0,0 | 0,0 | 6,1 | 11,0 |
| ***ALCAM*** | 2,0 | 0,6 | 1,9 | 0,5 | 2,4 | 1,1 | 3,0 | 1,6 | 1,3 | 0,6 | 0,8 | 0,8 | 1,4 | 0,4 | 1,5 | 0,4 | 0,4 | 0,2 | -0,1 | 0,2 | 0,3 | 0,5 | 0,9 | 0,6 |
| ***ANGPTL4*** | 9,5 | 1,5 | 5,4 | 1,0 | 6,6 | 2,0 | 7,2 | 2,7 | 10,2 | 0,5 | 8,8 | 0,4 | 7,9 | 0,5 | 8,8 | 1,4 | 13,8 | 0,2 | 10,9 | 0,6 | 7,1 | 1,3 | 7,3 | 1,6 |
| ***C5AR1*** | 8,2 | 0,4 | 9,8 | 1,6 | 11,1 | 2,8 | 7,7 | 3,8 | 7,8 | 0,4 | 9,6 | 1,8 | 9,9 | 2,3 | 10,8 | 2,0 | 10,8 | 0,6 | 7,2 | 0,3 | 8,4 | 1,5 | 7,8 | 0,3 |
| ***CD163*** | 0,0 | 0,0 | 18,1 | 3,7 | 14,7 | 6,0 | 12,0 | 8,3 | 3,5 | 8,5 | 17,2 | 1,9 | 9,4 | 10,3 | 15,1 | 8,1 | 3,2 | 8,6 | 3,1 | 7,7 | 2,6 | 6,9 | 2,6 | 6,8 |
| ***CD1A*** | 4,8 | 11,9 | 2,5 | 6,5 | 8,1 | 15,0 | 0,0 | 0,0 | 0,0 | 0,0 | 2,9 | 7,2 | 4,9 | 12,1 | 6,5 | 8,2 | 2,7 | 7,0 | 0,0 | 0,0 | 8,2 | 10,6 | 0,0 | 0,0 |
| ***CD1D*** | 11,5 | 0,7 | 12,4 | 0,6 | 12,1 | 1,6 | 11,5 | 1,6 | 11,8 | 0,7 | 12,1 | 1,0 | 11,9 | 0,5 | 12,7 | 1,4 | 16,9 | 1,2 | 11,3 | 0,9 | 12,6 | 6,1 | 12,4 | 0,7 |
| ***CD209*** | 3,9 | 9,6 | 2,1 | 5,7 | 3,3 | 9,3 | 3,4 | 9,1 | 3,5 | 8,5 | 0,0 | 0,0 | 5,1 | 7,9 | 5,5 | 6,8 | 0,0 | 0,0 | 0,0 | 0,0 | 2,6 | 7,0 | 0,0 | 0,0 |
| ***CD34*** | 1,3 | 0,2 | 11,2 | 1,6 | 12,1 | 3,1 | 10,8 | 2,9 | 13,1 | 9,4 | 10,7 | 1,8 | 10,9 | 1,5 | 11,9 | 1,5 | 11,7 | 0,9 | 8,7 | 4,5 | 11,7 | 1,7 | 8,4 | 3,7 |
| ***CD40*** | 8,2 | 0,2 | 10,1 | 0,9 | 10,7 | 0,6 | 10,4 | 0,4 | 12,3 | 0,6 | 12,3 | 1,1 | 12,3 | 0,8 | 10,4 | 2,1 | 15,9 | 0,8 | 14,5 | 1,0 | 13,6 | 1,3 | 13,4 | 1,1 |
| ***CD68*** | 7,0 | 0,3 | 4,3 | 0,3 | 4,6 | 0,2 | 4,2 | 0,5 | 4,6 | 0,3 | 4,8 | 0,1 | 4,2 | 0,2 | 4,4 | 0,4 | 4,6 | 0,2 | 5,2 | 0,7 | 5,4 | 0,6 | 5,5 | 0,8 |
| ***CD69*** | 10,8 | 0,3 | 13,0 | 1,8 | 13,8 | 2,5 | 11,2 | 1,7 | 11,1 | 0,7 | 12,5 | 2,3 | 12,3 | 1,7 | 12,2 | 1,1 | 16,5 | 2,1 | 10,3 | 0,8 | 12,7 | 1,8 | 11,2 | 0,5 |
| ***CD70*** | 7,7 | 0,3 | 9,2 | 0,9 | 10,7 | 2,5 | 9,0 | 2,5 | 7,1 | 0,3 | 10,5 | 3,1 | 9,8 | 1,8 | 9,4 | 3,4 | 10,9 | 0,6 | 8,1 | 0,7 | 11,3 | 3,3 | 8,1 | 0,5 |
| ***CD83*** | 14,2 | 0,6 | 13,1 | 0,7 | 13,5 | 0,7 | 13,9 | 0,8 | 17,4 | 1,2 | 17,3 | 1,7 | 15,9 | 0,9 | 14,2 | 4,2 | 16,9 | 0,4 | 17,8 | 1,1 | 17,1 | 0,9 | 16,9 | 1,0 |
| ***CD86*** | 0,0 | 0,0 | 0,0 | 0,0 | 4,1 | 7,7 | 6,7 | 8,4 | 0,0 | 0,0 | 5,0 | 7,9 | 2,8 | 6,9 | 6,2 | 6,3 | 4,5 | 7,7 | 2,4 | 5,8 | 4,7 | 8,0 | 0,0 | 0,0 |
| ***CD90/THY1*** | 10,2 | 0,2 | 5,8 | 0,8 | 5,3 | 1,0 | 5,7 | 1,6 | 4,6 | 0,3 | 4,4 | 0,5 | 4,8 | 0,4 | 4,3 | 0,3 | 4,3 | 0,1 | 2,9 | 0,3 | 3,2 | 0,8 | 3,6 | 0,2 |
| ***CDH5*** | 0,6 | 0,3 | 6,0 | 7,5 | 9,9 | 8,3 | 6,3 | 7,9 | 9,9 | 0,6 | 13,6 | 6,7 | 5,9 | 9,2 | 6,1 | 7,6 | 8,5 | 0,5 | 10,7 | 8,3 | 0,0 | 0,0 | 2,1 | 5,5 |
| ***CNN1*** | 12,3 | 3,5 | 11,9 | 0,9 | 12,8 | 1,9 | 10,8 | 4,9 | 6,0 | 0,2 | 9,1 | 0,6 | 9,4 | 0,9 | 9,3 | 1,2 | 7,1 | 0,3 | 6,7 | 1,7 | 6,5 | 1,0 | 8,1 | 1,0 |
| ***CSF1R*** | 0,0 | 0,0 | 12,2 | 8,4 | 16,9 | 1,2 | 9,8 | 9,2 | 12,5 | 9,7 | 17,1 | 1,1 | 13,6 | 6,7 | 10,8 | 7,6 | 16,7 | 1,2 | 13,6 | 6,8 | 14,9 | 6,7 | 12,8 | 8,8 |
| ***DDR2*** | 4,7 | 0,6 | 1,9 | 0,6 | 2,7 | 0,8 | 3,2 | 1,3 | 2,5 | 0,4 | 2,6 | 0,2 | 2,8 | 0,3 | 2,9 | 0,3 | 2,4 | 0,1 | 2,2 | 0,3 | 2,5 | 0,3 | 2,4 | 0,3 |
| ***ENG*** | 0,1 | 0,1 | 1,6 | 0,4 | 2,2 | 0,6 | 2,3 | 1,2 | 1,8 | 0,6 | 1,7 | 0,4 | 1,1 | 0,4 | 1,9 | 0,4 | 1,3 | 0,1 | 1,6 | 0,6 | 2,0 | 0,5 | 1,4 | 0,5 |
| ***EPCAM*** | 11,1 | 0,4 | 10,8 | 1,4 | 11,4 | 2,0 | 9,7 | 1,8 | 8,5 | 0,4 | 11,7 | 2,3 | 12,2 | 1,9 | 10,3 | 4,8 | 12,3 | 5,5 | 10,2 | 1,4 | 10,3 | 0,7 | 9,5 | 0,7 |
| ***FCER2*** | 7,6 | 0,3 | 10,5 | 2,6 | 10,4 | 5,5 | 7,2 | 3,5 | 7,2 | 0,5 | 13,1 | 8,3 | 10,3 | 2,7 | 9,6 | 3,3 | 9,0 | 6,4 | 8,2 | 0,5 | 11,9 | 9,3 | 8,1 | 0,3 |
| ***ICAM2*** | 4,3 | 0,3 | 11,4 | 0,7 | 11,4 | 0,3 | 11,1 | 0,2 | 14,3 | 0,7 | 13,3 | 0,7 | 12,0 | 0,9 | 11,3 | 1,9 | 14,4 | 0,8 | 6,6 | 7,2 | 13,7 | 1,0 | 13,1 | 0,6 |
| ***IL1R2*** | 0,0 | 0,0 | 9,6 | 1,1 | 10,3 | 0,6 | 9,8 | 1,0 | 10,9 | 1,4 | 13,7 | 1,3 | 12,9 | 1,3 | 12,8 | 1,3 | 12,2 | 0,5 | 7,1 | 7,8 | 12,2 | 5,4 | 13,0 | 0,9 |
| ***IL2RA*** | 0,0 | 0,0 | 2,4 | 6,2 | 2,1 | 6,0 | 2,3 | 6,0 | 0,0 | 0,0 | 2,6 | 6,3 | 2,6 | 6,3 | 4,4 | 5,5 | 0,0 | 0,0 | 0,0 | 0,0 | 0,0 | 0,0 | 0,0 | 0,0 |
| ***ITGA1*** | 7,7 | 0,8 | 6,1 | 0,7 | 6,5 | 0,9 | 7,1 | 0,8 | 4,9 | 0,6 | 4,4 | 0,6 | 4,6 | 0,6 | 5,1 | 1,0 | 5,1 | 0,3 | 4,1 | 0,3 | 5,5 | 0,5 | 4,7 | 1,1 |
| ***ITGA2*** | 3,3 | 0,5 | 5,1 | 0,6 | 5,6 | 1,5 | 6,0 | 1,3 | 4,5 | 1,2 | 4,5 | 0,9 | 4,8 | 1,3 | 4,4 | 0,8 | 4,1 | 0,1 | 3,1 | 0,7 | 3,7 | 0,9 | 4,2 | 0,7 |
| ***KRT18/***  ***AC107016,2*** | 6,4 | 0,3 | 7,7 | 0,4 | 8,4 | 1,3 | 8,7 | 1,9 | 4,7 | 1,5 | 6,4 | 2,2 | 4,7 | 0,6 | 7,1 | 2,1 | 7,0 | 0,6 | 6,7 | 0,6 | 6,2 | 0,4 | 5,2 | 0,9 |
| ***KRT5*** | 4,1 | 10,0 | 15,4 | 14,6 | 5,7 | 10,9 | 7,6 | 13,0 | 3,9 | 9,5 | 5,4 | 13,1 | 9,7 | 15,0 | 0,0 | 0,0 | 4,6 | 12,2 | 13,3 | 14,5 | 11,0 | 13,8 | 7,1 | 12,1 |
| ***KRT8*** | 8,0 | 0,4 | 9,4 | 1,1 | 10,1 | 1,8 | 8,8 | 1,6 | 7,2 | 0,3 | 7,2 | 0,7 | 8,5 | 0,7 | 9,1 | 1,8 | 9,5 | 0,3 | 5,6 | 0,2 | 7,1 | 1,6 | 7,9 | 0,4 |
| ***MYH10*** | 2,8 | 0,4 | 4,7 | 0,6 | 5,4 | 0,9 | 6,1 | 1,3 | 0,9 | 0,8 | 2,1 | 1,0 | 3,4 | 0,6 | 3,5 | 1,2 | 1,6 | 0,3 | 1,0 | 0,2 | 1,3 | 0,8 | 2,7 | 0,9 |
| ***MYH9*** | 0,2 | 0,5 | 2,8 | 0,4 | 3,2 | 0,8 | 3,5 | 0,6 | 1,3 | 0,7 | 1,7 | 0,7 | 2,6 | 0,3 | 2,7 | 0,9 | 1,5 | 0,1 | 0,4 | 0,4 | 1,2 | 0,4 | 1,9 | 0,5 |
| ***MYOCD*** | 0,0 | 0,0 | 10,3 | 7,1 | 11,2 | 7,0 | 2,4 | 6,2 | 6,6 | 1,5 | 10,5 | 1,3 | 11,1 | 1,1 | 10,6 | 2,2 | 12,1 | 0,5 | 10,3 | 0,2 | 9,9 | 1,6 | 11,2 | 2,1 |
| ***NOS3*** | 5,7 | 0,3 | 1,8 | 4,9 | 0,0 | 0,0 | 1,9 | 5,0 | 0,0 | 0,0 | 2,3 | 5,6 | 4,5 | 6,9 | 2,1 | 5,6 | 0,0 | 0,0 | 0,0 | 0,0 | 0,0 | 0,0 | 0,0 | 0,0 |
| ***PECAM1*** | -1,6 | 0,2 | 8,1 | 0,4 | 9,3 | 1,8 | 8,7 | 3,0 | 6,0 | 0,5 | 8,2 | 1,5 | 8,5 | 1,7 | 9,4 | 1,9 | 10,9 | 1,1 | 6,7 | 0,7 | 8,8 | 1,3 | 6,8 | 0,5 |
| ***RETN*** | 6,9 | 0,2 | 9,7 | 2,7 | 7,4 | 5,5 | 6,8 | 3,6 | 6,3 | 0,3 | 5,2 | 4,0 | 9,8 | 3,1 | 12,2 | 3,3 | 9,0 | 6,4 | 7,3 | 0,8 | 10,8 | 3,6 | 7,3 | 0,6 |
| ***S100A4*** | 17,2 | 0,7 | 9,7 | 2,2 | 9,4 | 1,9 | 8,6 | 1,5 | 12,8 | 0,7 | 8,7 | 0,5 | 9,4 | 0,8 | 9,8 | 0,7 | 10,7 | 0,3 | 9,6 | 2,3 | 8,8 | 1,6 | 9,5 | 1,0 |
| ***S100A8*** | 18,6 | 0,6 | 13,6 | 9,4 | 17,3 | 11,1 | 20,3 | 2,6 | 17,6 | 0,9 | 21,3 | 3,2 | 21,5 | 2,4 | 17,9 | 12,9 | 12,6 | 11,9 | 18,9 | 1,4 | 18,4 | 8,1 | 18,8 | 0,8 |
| ***SELP*** | 12,6 | 1,7 | 0,0 | 0,0 | 0,0 | 0,0 | 0,0 | 0,0 | 8,3 | 9,1 | 0,0 | 0,0 | 5,2 | 8,1 | 2,2 | 5,9 | 8,6 | 8,1 | 2,7 | 6,7 | 2,4 | 6,4 | 11,4 | 7,8 |
| ***SMTN*** | 10,8 | 0,3 | 4,4 | 5,5 | 8,5 | 5,3 | 1,5 | 4,1 | 3,4 | 5,3 | 7,1 | 5,5 | 9,0 | 4,4 | 5,8 | 5,5 | 0,0 | 0,0 | 7,9 | 3,9 | 8,9 | 4,0 | 7,2 | 5,0 |
| ***TEK*** | 4,5 | 0,9 | 9,8 | 1,5 | 10,3 | 2,3 | 8,1 | 6,1 | 7,5 | 0,6 | 7,5 | 1,0 | 7,4 | 1,0 | 7,5 | 0,3 | 6,4 | 0,4 | 8,6 | 0,3 | 10,0 | 1,5 | 8,5 | 2,1 |
| ***TNFRSF8*** | 5,8 | 9,1 | 12,2 | 1,2 | 12,0 | 2,4 | 6,5 | 6,3 | 10,7 | 0,4 | 12,1 | 9,1 | 10,5 | 0,5 | 9,6 | 3,1 | 13,1 | 1,2 | 10,1 | 12,9 | 12,0 | 8,9 | 10,7 | 5,6 |
| ***VCAM1*** | 7,2 | 1,2 | 7,1 | 1,3 | 7,6 | 2,3 | 7,9 | 2,4 | 6,8 | 1,4 | 7,3 | 1,8 | 7,8 | 1,1 | 5,9 | 2,4 | 10,0 | 0,2 | 8,0 | 0,9 | 10,3 | 2,9 | 9,0 | 1,2 |
| ***VWF*** | -0,8 | 0,1 | 11,2 | 0,6 | 11,3 | 0,4 | 10,5 | 0,6 | 14,6 | 0,7 | 12,1 | 0,5 | 12,6 | 0,2 | 12,5 | 0,3 | 12,0 | 5,3 | 12,1 | 0,7 | 11,9 | 0,6 | 11,7 | 0,6 |
